# The influence of belief in disaster myths on first responders' decision-making in emergencies and disasters — A scoping review

**DOI:** 10.3389/fpubh.2026.1801679

**Published:** 2026-07-02

**Authors:** Amnon Alkalai Tavori, Hadas Marciano, Bruria Adini

**Affiliations:** 1 Emergency & Disaster Management Department, School of Public Health, Gray Faculty of Medical and Health Sciences, Tel Aviv University, Tel Aviv, Israel; 2 Stress and Resilience Research Center, Tel-Hai University of Kiryat Shmona in the Galilee, Kiryat Shmona, Israel; 3 The Institute of Information Processing and Decision Making (IIPDM), University of Haifa, Haifa, Israel; 4 ResWell Research Collaboration, Tel Aviv University, Tel Aviv, Israel

**Keywords:** decision-makers, emergencies and disasters, first responders, myths, public behavior

## Abstract

**Introduction:**

The ability to anticipate public behavior shapes first responders' actions during emergencies. Discrepancies between empirically grounded knowledge of public behavior and persistent disaster-related myths held by first responders may lead to misaligned decisions, placing human lives at risk.

**Methods:**

A systematic scoping review mapped the existing evidence on how belief in myths influences the decision-making of first responders (e.g., police/fire services) during the emergency response phase. The review followed the Population-Concept-Context framework and is reported according to PRISMA-ScR guidelines. The search strategy included three stages: a systematic search of electronic databases (Scopus, PubMed, JSTOR, ScienceDirect, and Google Scholar), manual screening of reference lists, and forward citation tracking from 2020 onward. Of the 859 sources identified, 21 met all inclusion criteria and were included in the review.

**Results:**

Findings demonstrate that myths, such as assumptions of public panic, looting, and social breakdown, exert direct and indirect influence across multiple levels of the emergency system. These beliefs are embedded in organizational culture, training, procedures, policies, and communication practices, often leading to centralized, security-oriented, and command-and-control responses. Such approaches divert resources from life-saving actions, suppress civic initiatives, restrict information flow, and undermine trust, ultimately reducing response effectiveness and exacerbating harm. These patterns recur across disaster types and contexts, indicating that decision-making failures stem less from disaster characteristics than from myth-based assumptions.

**Conclusions:**

The study concludes that improving emergency response requires a paradigm shift toward evidence-based models that recognize the public as a resource, promote decentralized coordination, support civic participation, and prioritize transparent, empowering communication.

## 1 Introduction

The ability to predict public behavior is a critical component of emergency management, as these expectations often shape the actions of first responders arriving at areas affected by disasters. Expectations may influence actual behavior; for example, an extended hand may be interpreted as a greeting and thus elicit cooperative behavior, whereas the same gesture may elicit avoidance or defensive responses if perceived as threatening (1). Similarly, first responders who anticipate panic among occupants in an event hall in response to a fire alert may attempt to maintain order by withholding clear and explicit warnings, thereby increasing the risk to human life (2).

Interactions between first responders and the public strongly influence behavioral responses during emergencies and disasters (3). Discrepancies in the content, quantity, or credibility of information may compromise public trust in authorities and reduce compliance with official instructions (4). Consequently, the response strategies adopted by authorities may elicit behavioral patterns that do not necessarily facilitate an orderly or effective emergency response (5). Such dynamics and inaccurate expectations about public behavior may generate persistent, self-perpetuating deficiencies in response effectiveness and risk reduction. This is particularly unfortunate, since members of the public can represent a substantial operational resource during a disaster response when used correctly (6).

A disaster myth is defined as a scientifically refuted belief concerning human behavior in disaster contexts (7). Specifically, many general expectations about human behavior during disasters contradict empirical findings of orderly, adaptive patterns of response. Such misconceptions have been identified across a wide variety of disaster types and cultural contexts (2, 7, 8). For example, the assumption that emergencies commonly produce widespread panic, looting, or social disorder has been repeatedly refuted by empirical evidence (9). Another prevalent but inaccurate assumption is that much of the population becomes stunned or unable to function effectively during emergencies (10, 11). Indeed, a synthesis of existing research in the field identifies approximately 70 distinct disaster-related myths concerning human behavior during emergencies and disasters (7).

The operational importance makes it crucial for emergency-management professionals to have an accurate, evidence-based understanding of public behavior during disasters. However, studies indicate that belief in disaster myths is widespread even among professionals (11). For example, research conducted among municipal emergency managers in Japan identified levels of myth endorsement comparable to those of the general public (8). Similar findings of equivalent or only slightly lower levels of belief in myths among professionals compared to the general public emerged from studies involving police officers, municipal employees, and sports event stewards in the UK (2). Comparable trends have also been identified among students in emergency management, nursing, and related fields in both the United States and Italy (7).

A recent Israeli study concluded that first responders, including police, emergency medical services, and fire services, believed in some myths more strongly than the general public, and to the same degree in others (11). Interestingly, studies demonstrate that myth endorsement is present across societies worldwide, although the prominence of specific myths varies by cultural context (11). For example, belief in increased crime during disasters is widespread in Japan, while belief in public panic during disasters is less common (8). In contrast, other societies feature fear of panic as central to disaster planning and procedures, whereas the myth of increased crime is less dominant.

The real problems with believing in disaster myths lie not only in their prevalence but also in their influence on decision-making and operational strategies. For example, studies of Hurricane Katrina reported that myths relating to crime, panic, and social breakdown contributed to operational delays and misallocation of resources, and reduced the efficacy of response efforts (12).

Despite the importance of this issue, existing research into the influence of myth-related beliefs on decision-making among responders remains limited and fragmented. Much of the available knowledge is derived from analyses of past events, official investigation reports, and theoretical literature, rather than systematic empirical studies. Moreover, relevant insights are distributed across multiple disciplines, including emergency management, psychology, sociology, and communication, and vary widely in their conceptual frameworks, terminology, and analytical foci. Frequently, explanations for behavioral patterns in disasters rely on psychological or cultural assumptions that are not empirically validated.

In the absence of a coherent, data-driven body of knowledge, there is a need to systematically collect, map, and synthesize existing findings across sources and disciplines to develop a comprehensive understanding of how belief in disaster myths influences responder decision-making. A Scoping Review methodology is particularly appropriate for this purpose, as it enables us to identify the breadth of available knowledge, integrate findings from diverse sources, delineate conceptual boundaries, and recognize research gaps. This will support the development of future empirical studies and contribute to a deeper understanding of this complex, multidimensional phenomenon.

The aim of the current Scoping Review is to review and map existing research on the influence of widespread belief in myths on the decision-making processes of first responders in disaster and emergency situations. To this end, we examine how disaster-related myths influence disaster responses and the operational course of action. Specifically, we focus on myths that have a concrete, documented impact due to a discrepancy between prevalent beliefs and empirical research on human behavior in disasters. Our findings will provide the basis for recommendations for developing expertise and strengthen professional responses in emergencies and disasters.

## 2 Methodology

### 2.1 Study design

This systematic scoping review was conducted using the PCC (Population–Concept–Context) framework (13) and is reported in accordance with the PRISMA-ScR guidelines (14).

The search strategy comprised three consecutive, complementary stages, designed to ensure broad, relevant, and in-depth coverage of the literature on the influence of belief in myths on the decision-making of first responders in disaster events. The review was conducted systematically in accordance with a protocol developed by the research team prior to the study. One researcher conducted an initial screening using predefined inclusion and exclusion criteria (Table 1), while two additional researchers reviewed all inclusion and exclusion decisions to ensure quality and consistency.

**Table 1:** Inclusion and exclusion criteria.

Criterion	Inclusion	Exclusion
Content relevance & substantive contribution	Sources that examine, describe, or meaningfully discuss the influence of myths on decision-making, event management, or response strategies used by first responders during emergencies or disasters. Included were sources including substantial discussion in the results, discussion, or conclusions sections, as well as sources providing sufficient information to support the mapping and understanding of how myths affect decision-making, management, and response approaches	Sources in which the discussion of myths or beliefs is minimal, marginal, or purely declarative, or which do not provide content relevant to understanding how myths influence decision-making, management, or response strategies during emergencies or disasters
Population	Studies involving first responders: police, firefighters, EMS, and rescue personnel. Also included were sources in which first responders appear as part of a broader emergency group (e.g., emergency authorities, incident management teams), provided the source explicitly states that first responders are part of the studied group. Studies focusing on a single emergency agency (e.g., police only) were also included	Sources focusing on populations that are not first responders, or where first responders are not explicitly included in the study population. Examples include civilians, communities, untrained volunteers, government officials, politicians, or senior administrative personnel not directly involved in emergency response decision-making
Concept	Sources addressing the influence of beliefs or myths on decision-making, management, response strategies, or operational handling during the emergency response phase. Included were sources describing direct or indirect influences of myths, provided the connection to decision-making or management is explicitly stated	Sources discussing impacts unrelated to decisions, management, or operational response during emergencies. Sources addressing only recovery, rehabilitation, preparedness, or mitigation, without relevance to the response phase, were excluded
Myth-related discussion	Sources that provide a clear and meaningful description of how myths or inaccurate beliefs influence decision-making, event management, or response strategies in emergencies or disasters	Sources addressing beliefs or myths—including those held by first responders—without meaningful discussion of their influence on decision-making or response management in emergencies
Theoretical alignment	Sources using the term *myth* according to the accepted definition in disaster research—a gap between empirical evidence and widely held public or professional beliefs (7). Sources using the term *disaster* according to the WHO definition (33)	Misuse of the terms “*myth”* or “*disaster”*, or use as inconsistent with the definitions provided by Alexander (7) or the WHO (33)
Publication type	Peer-reviewed academic articles, gray literature with research or applied value, and articles published in edited volumes. No year limits were applied	Full books (monographs) and publications in languages other than Hebrew or English
Full text availability	Sources for which the full text was accessible and could be used for analysis and synthesis	Sources available only as abstracts, partial texts, or inaccessible full texts

The first stage involved a systematic search in electronic databases, including Scopus, PubMed, JSTOR, ScienceDirect, and Google Scholar. A dedicated search string was developed for each data engine, based on three key concepts: disaster myths, first responders, and decision-making. Search terms included,: “disaster myths,” “emergency myths,” “false beliefs,” “misconceptions,” alongside “first responders,” “emergency personnel,” “police officers,” “firefighters,” “paramedics,” and “emergency medical technician,” and terms such as “decision making,” “judgment,” “risk perception,” “situational awareness,” and “response behavior.” The terms were combined using the Boolean operators (AND/OR) to create broad and precise coverage.

The second stage of the review involved a manual examination of the bibliographies of all articles included in the first stage. The examination focused on identifying additional sources relevant to the research question. Sources other than books or book chapters featured in more than one bibliography (two or more reference lists) were read in full and were then subjected to predefined inclusion and exclusion criteria (Table 1). Sources with at least one term related to myths, behavior, public response, panic, looting, first responders, or decision-making in the title were subjected to a staged screening process, with inclusion and exclusion criteria applied at each step: title review, abstract screening, and full-text assessment. This was designed to ensure methodological consistency across all search methods. In the third stage, all articles identified in the previous stages were examined through forward citation searching in Google Scholar to identify recent studies that had cited them. The search used a string containing the terms myths, first responders, and impact OR influence. All publications published from 2020 onward that met these criteria were included to identify emerging developments or new findings on the topic.

The data charting process was carried out in two stages to ensure systematic and collaboratively reviewed analysis. First, one researcher compiled data from all summary forms; the findings were then reviewed and discussed with two additional researchers to reach consensus on the central themes. In the second stage, these themes were re-examined through focused reading of the relevant articles to gather additional references and refine the synthesis by applying an integrative-thematic approach. The process was iterative, involving repeated examination, feedback, and joint revisions by all three researchers to ensure coherence and rigor.

The first stage systematic search identified a total of 606 items across all examined search engines. A close assessment of these items against the inclusion and exclusion criteria yielded 14 eligible articles.

A manual search of the bibliographic lists of these articles conducted in the second stage of review yielded 934 items, of which 71 items were selected for further assessment according to the eligibility criteria. These articles included items that appeared more than once across the bibliographic lists (14 recurrent items) and items identified through keyword searches in the bibliographic titles. The second stage screen yielded five additional articles that met all eligibility criteria. One additional item was identified through manual searching, bringing the total to 6 eligible articles, for a final total of 20.

Forward citation searching on these 20 articles in the third stage of review identified citations by 3,833 items. Screening according to the search criteria defined for this stage identified 181 items as requiring further assessment. Examination of these items according to the staged eligibility criteria identified one additional article as eligible for inclusion.

**Figure 1:**
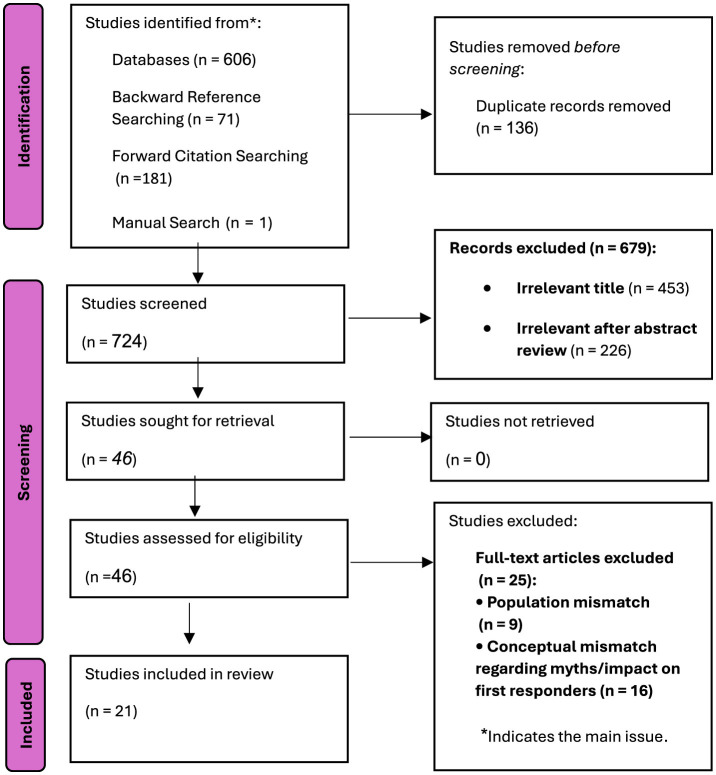
PRISMA flow diagram of article selection.

Overall, the three search stages yielded 859 items, which met the initial search criteria and therefore required examination according to the inclusion and exclusion criteria. Subsequent assessment through the staged screening excluded articles for various reasons (Figure 1) and yielded 21 articles that met all inclusion criteria and were included in the review.

## 3 Results

Following the PRISMA-guided identification, screening, eligibility assessment, and selection process presented in Figure 1, 21 studies met the inclusion criteria and were included in the review. Detailed characteristics of these studies are provided in Supplementary Table S1.

A total of 16 peer-reviewed journal articles, 3 gray literature sources, and 2 book chapters/edited volume contributions were included in the review. Methodologically, the corpus comprised 13 theoretical/conceptual papers, 6 qualitative studies, 1 quantitative study, and 1 mixed-methods study.

Only a single study empirically examined the relationship between belief in disaster myths and preferred response strategies among operational personnel (police officers, stewards, safety professionals, and members of the public), directly comparing individuals who endorsed disaster myths with those who did not (2). The remaining studies offered descriptive analyses, conceptual discussions, or qualitative evidence (e.g., 6, 17, 18, demonstrating how myth-based assumptions influence decision-making, operational behavior, and emergency management structures. Some studies proposed alternatives to dominant command-and-control models. However, there was no empirical examination of whether the absence of myth-belief translates into different behavioral choices in practice.

**Figure 2:**
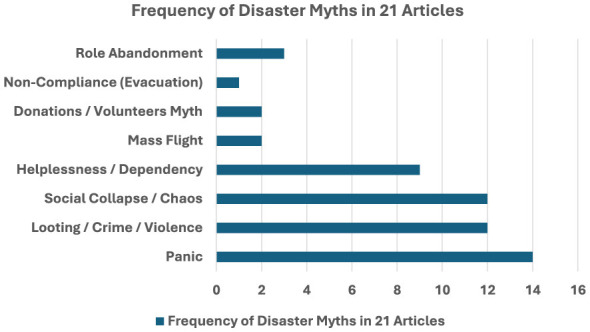
Disaster myths reported as affecting emergency decision-making (*n* = 21 articles).

Findings from the 21 included sources provide evidence for the significant influence of disaster myths on decision-making processes and operational practices within emergency response organizations, albeit to varying degrees. The central disaster myths identified in the reviewed literature, along with the studies that refer to each, are presented in Figure 2. These myths are shown to influence first responders' decision-making during disasters and emergencies. The diagram indicates the number of studies addressing each myth, with a maximum score of 21. Our results indicate that the most frequently discussed myth is the panic myth, with 14 studies examining how belief in public panic affects first responders' decision-making. Despite specific differences, we can detect a common pattern: all the myths portray the public as problematic or unpredictable. This perceived pattern of public behavior during disasters consistently influences decision-making, management approaches, and operational responses in emergency and disaster contexts (15–17).

## 4 Discussion

### 4.1 Summary of findings—Themes and sub-themes

Table 2 presents the main themes and sub-themes regarding the influence of myths on first responders' decision-making that emerged from the integrative synthesis of the items included in the review.

**Table 2:** Themes and sub-themes.

No.	Theme	Sub-theme	Section reference
1	Circles of influence and modes of influence of myths	Axes of influence—direct and indirect	4.2.1
		Indirect platforms through which disaster myths shape emergency response decision-making	4.2.2
		Circles of influence of myths and hierarchy of influence	4.2.3
2	The influence of myths on decision-making and operational approaches in disaster management	Preference for a command-and-control model	4.3.1
		Preference for managing the event as a security rather than a civilian incident	4.3.2
		Task prioritization and resource allocation	4.3.3
		Use of enforcement measures toward the public	4.3.4
		Suppression of civic initiatives and exclusion from planning processes	4.3.5
		Controlled information management and communication	4.3.6
		Decision-making based on media-driven images	4.3.7
3	Decision-making characterized by the influence of specific myths		4.4
4	Findings from an Integrative perspective on disaster events influenced by myths		4.5
5	Recommendations for addressing the influence of myths on first responders' decision-making during disasters		4.6

### 4.2 Circles of influence and modes of influence of myths

#### 4.2.1 Axes of influence—Direct and indirect

Myths about public behavior during disasters influence decision-making regarding actions and the management of emergency and disaster events both directly and indirectly. A direct effect is seen when first responders or other decision-makers involved in emergency and disaster responses base their decisions on the possibly erroneous expectation that a disaster myth is real. This can result in operational choices that are misaligned with empirically documented patterns of human behavior and may result in ineffective or undesirable response actions (2, 3, 18).

A more indirect influence is caused by myths becoming embedded over time within processes, with consequent effects on emergency and disaster management, for example, within policy (19, 20), doctrine, or operational procedures (15, 21, 22). This dictates the actual framework within which first responders operate, thereby affecting their conduct and decision-making during the event (12, 15, 16, 23, 24).

#### 4.2.2 Indirect platforms through which disaster myths shape emergency response decision-making

Indirect forms of influence are reflected across multiple platforms, where myths become an inherent part of emergency systems. These platforms include:

##### 4.2.2.1 Foundational perceptions and operational doctrines

Myths shape the way disasters are defined and understood within professional perspectives and operational doctrines. For example, whether disasters are perceived as security-related or humanitarian, or as threatening or manageable. These definitions dictate operational models, defined responsibilities among organizations, and operational priorities (12, 20, 21).

##### 4.2.2.2 Organizational culture and professional values

Embedded beliefs about “public behavior” become internal norms that shape attitudes, professional identity, and modes of operation. Organizational culture is formed around perceptions of control, hierarchy, and command—or alternatively, around perceptions of cooperation, trust, and resilience (6, 15, 25).

##### 4.2.2.3 Procedures, protocols, and operational models

Responses to deeply embedded myths are articulated as formal procedures: access restrictions, evacuation patterns, prioritization orders, information delivery, and public engagement procedures. These procedures shape the operational context of first responders and the possibilities they perceive as feasible (9, 10, 15, 17, 22).

##### 4.2.2.4 Professional training and skill development

The beliefs that emergency personnel acquire explicitly or implicitly during training programs shape their situation awareness and decision-making processes. When training is based on myths, it entrenches operational practices that do not align with empirical understanding of human behavior (15, 25).

##### 4.2.2.5 Emergency exercises and simulations

Emergency exercises are based on underlying assumptions; when these assumptions are mythological, training reinforces them and grants them operational legitimacy. Exercises are one of the primary mechanisms through which beliefs become mandatory practices. For example, the lack of dedicated training in working with volunteers and emergent citizen groups creates uncertainty among first responders. As a result, they may fail to utilize valuable civilian responders in real time, thereby perpetuating actual organizational exclusion (6, 23).

##### 4.2.2.6 Disaster response plans

Myths about public behavior during emergencies influence the design of response plans by embedding models and operational perceptions based on incorrect assumptions. These plans then shape the response and decision-making of emergency organizations and first responders during the event (6, 15, 17, 19).

##### 4.2.2.7 Institutional structures and inter-organizational interfaces

Myths shape interactions among organizations, including the division of responsibilities, authority levels, coordination, information flows, and decision-making mechanisms. These structures determine what individuals within organizations are allowed to decide, how much judgment they can exercise, what information is available to them, and which courses of action are considered legitimate during emergencies (6, 12).

##### 4.2.2.8 Public policy and normative regulation

Legislation, regulations, and policy documents formulated based on assumptions rooted in myths affect emergency organizations and their decision-making processes (24).

#### 4.2.3 Circles of influence of myths and hierarchies of influence

There are extensive literature reports that myths about public behavior during disasters influence decision-making across the entire response chain—from the system, through the organization, and down to the first responder (12, 15, 20, 25).

Direct influence may occur both at the upper system level, when policymakers and senior commanders make strategic decisions based on myth-based assumptions (26), and at the level of the responder in the field, when the situation and public behavior are interpreted through the lens of the myth and action is taken accordingly (2). In such cases, the myth directly shapes risk assessment, the selection of the course of action, and operational priorities (2).

In contrast, indirect influence occurs when myths are embedded in advance within the operational mechanisms of organizations and institutions responsible for the initial response (15–17, 21, 23), or alternatively within the entire emergency management system (12, 20, 22, 24).

**Figure 3:**
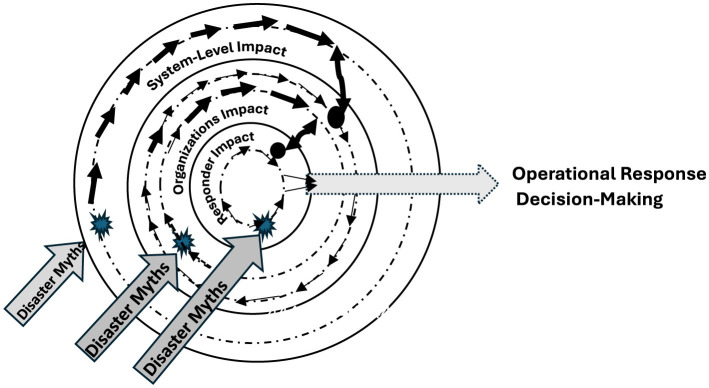
Multi-level influences of disaster myths on first responders' decision-making. The model illustrates the concomitant influence of disaster myths on three levels: the individual, the organization, and the emergency management system. Professional perceptions and organizational processes at each level are shaped by these myths. These processes frame the operational environment and shape the individual responder's real-time decision-making, ultimately determining how the response is delivered. Accordingly, the influence of disaster myths on responders is both direct, through their own beliefs, and indirect, through the influence of organizational and system-level factors.

When integrating all the findings, a picture emerges of three circles of influence that operate like ripples: systemic perceptions shape organizational responses; organizations shape the thinking patterns, training, and preparation of their personnel; and ultimately, first responders operate in real-time within a conceptual and organizational framework shaped by those perceptions. The multi-level pathways through which disaster myths shape decision-making are presented in Figure 3.

### 4.3 The influence of myths on decision making and operational approaches in disaster management

#### 4.3.1 Preference for a command-and-control model

The belief that the public tends toward chaotic behavior, panic, or helplessness and dysfunction reinforces the tendency to adopt emergency management command-and-control models. These may be characterized as centralized, hierarchical, “top-down,” bureaucratic, influenced by military logic, rigid, and inflexible (9, 21, 22, 27). According to this view, routine civilian institutions are perceived as incapable of effectively managing emergencies, as their fragile structures and inefficiencies are assumed to intensify during crises. This assumption holds that civilian institutions must be strengthened or replaced by external organizations, and that the most effective organizations are those that operate with military-like characteristics, such as the police and fire services. Consequently, other organizations are also expected to act in this way in order to function effectively during emergencies, while existing social structures, such as volunteer organizations or emergent groups formed *ad hoc* during disasters, are regarded as largely irrelevant (27, 28).

Unfortunately, the literature suggests that a centralized, hierarchical, and militarized approach is ineffective and tends to fail. One reason is the rigidity of the command-and-control model, which promotes interorganizational conflict and impedes interorganizational communication (21). Such models may not be applicable to community settings and constitute a flawed basis for emergency planning (10). The command-and-control approach gives the police and military exclusive authority for providing assistance and oversight, while the public is viewed as subordinate, passive, and lacking influence. Decisions are made to place control and authority in central management hubs, allow only professional bodies to make decisions, and reject local or community initiatives. This perception is sustained, despite evidence that even informal civilian responses provide substantial assistance in coping with disasters and in recovery processes (6, 15, 16, 19, 22, 27).

Another issue is that centralized action based on hierarchical command-and-control slows response time, hinders coordinated efforts, and constrains flexibility and adaptation to rapidly changing realities (10, 16, 22). In addition, this style fosters a tendency toward bureaucratic overreaction (27).

#### 4.3.2 Preference for managing the event as a security rather than a civilian situation

Under the assumptions of loss of control, looting, and chaotic behavior, the disaster may be defined as a security situation, leading to the deployment of armed forces, activation of national guard units, and implementation of military-style operational patterns (2, 24, 25).

The trend of militarizing disaster responses is reflected in the use of military forces to perform policing functions and impose control and order, rather than focusing on humanitarian assistance (24). Operational directives emphasize “restoring control,” and employ military language, such as “front line” and “control”, with associated imagery that reinforces the perception that the public itself constitutes a source of threat (12). In parallel, there is pressure to mobilize military forces for emergency missions and to grant them broad civilian authority, including the use of force against the population (24).

#### 4.3.3 Task prioritization and resource allocation

When authorities believe that the public tends toward looting, anarchy, or panic, they make decisions that prioritize internal security, control, and the preservation of public order (12, 18).

In such situations, resources are diverted from search and rescue, food distribution, and basic assistance to affected populations toward maintaining public order. Rather than focusing on rescue activities and assisting the population, forces are diverted from humanitarian relief to security missions, including maintaining curfews and controlling movement. This wastes essential resources on theoretical problems (12, 18, 27, 29), such as the necessity to escort and safeguard supply chains. This “escort-first” policy may delay the delivery of crucial food, water, and sanitation services until escort approval is obtained, thereby unnecessarily prolonging human suffering (24). Operational efforts may also focus on identifying “internal threats” and deploying police personnel and soldiers instead of locating survivors and transporting essential supplies (12).

#### 4.3.4 Use of enforcement measures directed toward the general public

According to a common perception, a collective response to disasters is characterized by rioting and criminal behavior, such as looting, stemming from the collapse of the “thin veneer of civilization” and the exposure of the “barbarism” presumed to lie beneath (30).

Based on this assumption, once the masses sink into a primitive and irrational psychological state, the use of extensive legal and administrative force, and at times even coercive measures, is considered a justified means of restoring social order (12).

The assumption is that when people reach such a state, they must be controlled with forceful measures (24). As a result, operational approaches involve the use of legal powers to impose order and control, including arrests, detentions, use of force, and entry into private premises (24). Actions include closing areas, blocking access to disaster zones, and introducing checkpoints, cordoned areas, enhanced identification checks, and restrictions on movement of survivors and their family members, as well as postponing the return of evacuees to their homes (19, 25).

The logic underlying such decisions and actions is that centralized control is required to prevent problems of order and security and to enable a more efficient response (2). In extreme cases, the declaration of martial law may even be considered (25). Emphasis is placed on enforcing strict laws, based on the assumption that otherwise the public will not exhibit “proper” behavior (27).

#### 4.3.5 Suppression of civic initiatives and exclusion from planning

Limiting responsibility to professional or quasi-military personnel reflects the popular perception of civilian organizations' inability to cope with emergencies and the belief that spontaneous civic initiatives may increase vulnerability (19, 23, 27).

These assumptions translate into suboptimal operational practices during emergencies, including delayed recognition of citizen initiative and contribution and the removal of bystanders from the scene out of fear that they will interfere with rescue operations, rather than their controlled integration into rescue and assistance efforts (16). Citizens themselves are excluded from planning and response, while the community's ability to self-organize is ignored, resulting in the loss of a significant human resource in the field (25). Community organizations and volunteers are excluded from emergency plans, and tensions sometimes arise between the public sector, the formal third-sector volunteer organizations, and the “fourth sector” of emergent, unaffiliated volunteers (23).

Emergency plans are built around the government as the sole “service provider” and therefore do not invest in strengthening community and civic capacities, such as local search-and-rescue. This planning pattern is common even when community and civic entities constitute the primary source of initial operational capability during disasters (27). In practice, the professionalization of emergency management, which was originally intended to encourage civic participation and reduce the military approach, is increasingly dominated by security institutions and narrow definitions of expertise (2, 22). This results in an emergency management system that is professionally weakened and emphasizes top-down action and planning without public integration (6).

Excluding civilian response and spontaneous public behavior from emergency planning leads to failures in anticipating and preparing for potential disaster scenarios. As a result, responders and institutions are often unprepared for the actual public response to disasters (6, 23). For example, the fact that large numbers of casualties are often transported from the scene to the nearest hospital by members of the public requires preparation for this type of evacuation, including hospital operations, traffic management, and public order management (16). Many emergency plans do not recognize any informal civilian response, which may then become a source of conflict (6). Notably, in the absence of expectations and guidance, uncoordinated volunteers may indeed become a burden (10). For example, different teams may check the same piles of earthquake debris. This can be remedied by appointing leaders, dividing work groups, and connecting to a central hub using some communication method (9).

#### 4.3.6 Controlled information management and communication

The belief that the public will panic leads authorities to make decisions to restrict the flow of information to the public, postpone the release of warnings, or conceal data about the scale of the disaster (2, 3, 12, 27). The fear that panic will cause greater harm than the disaster itself is referred to as “elite panic” in disaster research (26). This term refers to a situation in which those in positions of authority, from policymakers to senior managers, fear the possibility of chaotic mass response and therefore take steps to conceal information, enforce communication control, or use stringent security measures to prevent such reactions (2, 18).

The rise of social media has fundamentally altered the information environment in which authorities and decision-makers operate. Rapid, decentralized, and real-time information flows make it more difficult to control information and shape public narratives, while simultaneously increasing concerns regarding the influence of incomplete, misleading, or inaccurate information (misinformation/disinformation) on public perceptions and behavior (31). Accordingly, contemporary manifestations of elite panic extend beyond withholding information and communication control alone and include actions aimed at controlling information flows, shaping public narratives, and addressing the perceived effects of misinformation and disinformation on public responses during crises and disasters. Nevertheless, relatively little is known about how social media environments influence contemporary manifestations of elite panic. Future research should examine these relationships more directly, particularly given findings suggesting that assumptions regarding the effects of social media on public reactions are not always empirically supported (32).

The phrase “don't shout fire in a crowded theater” has become symbolic of the fear of panic, despite findings that clear and explicit warnings may, in fact, save lives by prompting people to evacuate quickly (9). A policy of selective communication allows the dissemination of “reassuring information” only, while “sensitive” information is retained by authorities under the belief that they “know better” and are presumably immune to the “pathological” reactions attributed to the public (2, 26).

A typical example followed the radiation leak at the Fukushima Daiichi power plant in March 2011, when the Japanese government delayed publishing radiation-dispersion forecasts, claiming that the information could provoke panic, even though the action endangered residents and exacerbated their situation. In May 2011, the government apologized for this decision. This case illustrates how fear of panic and the ensuing decision to refrain from effective risk communication actually endanger the public (18).

Concealing information under the rationale of “preventing panic” undermines the public's ability to engage in protective actions and erodes trust in authorities (3, 22).

#### 4.3.7 Decision-making based on media-driven images

Decision-makers tend to rely on media reports and images of anarchy and looting rather than on field data (12). The resulting decisions may then be based on fear, public pressure, and the desire to “demonstrate control of the event,” rather than on factual data and professional risk assessment (28).

While intended to “maintain order,” decisions to restrict journalists' access to disaster zones, except for escorted and pre-planned encounters, may actually reinforce myths (24). Notably, the literature reviewed did not identify more recent empirical studies that directly examine this issue, particularly regarding the role of smartphones and social media in shaping these dynamics.

### 4.4 Decision-making characterized by the influence of specific myths

It is important to note that the myths themselves appear universal across cultures. However, the degree to which they are believed and their intensity vary across national contexts (11). Accordingly, the practical manifestations in the field may differ as well. This highlights the importance of understanding the association between different prevalent myths and the concrete, expected decision-making patterns of first responders. The key actions and decisions based on each myth are presented in Table 3.

**Table 3:** The unique influence of major myths on first responders' decision-making across different categories.

Myth/influence category	Centralized control	Treating the event as a security situation	Resource allocation	Enforcement and suspicion toward the public	Suppression of civic initiatives and exclusion from planning	Lack of preparedness for public behavior	Managed information flow and controlled communication
Panic							
Helplessness							
Looting and increased crime							
Social breakdown							
Role abandonment							
Mass flight and evacuation							
Donations and shipments							
Organizational paralysis							

The darker pathways represent the central and direct influences of each myth, whereas the lighter color indicates additional influences that accompany the myth or coexist with other myths.

A fear of panic may cause responders and senior officials to delay issuing a warning, even when the risk is tangible. Critical information is withheld, and the public loses the ability to prepare or evacuate in time (2, 3, 12). Belief in panic is also used as a basis for justifying centralized and closed management structures, and the establishment of military-like hierarchical command frameworks, from which local initiative and civic participation are excluded (28).

The mass flight myth is sometimes discussed alongside the panic myth and is occasionally treated as an independent entity (12). The fact that the public tends not to evacuate immediately but instead seeks information results in delayed evacuations, along with traffic congestion and operational consequences (12). This also creates a perceived need to control the public through strict instructions and carefully managed messages (10).

The influence of the helpless-victim myth may cause first responders and event managers to treat spontaneous citizen initiatives as an obstruction rather than a useful resource (6). The assumption of helplessness positions the public as dependents waiting for assistance from authorities. This strengthens institutional superiority, a lack of partnership, centralized control, and even the need for security-oriented management (2, 16). When citizens act nonetheless, their activity is perceived as chaotic and unmanaged (6).

Interestingly, the looting myth has unique effects. A central characteristic is the diversion of resources toward preventing looting, and the deployment of enforcement and suspicion toward the public. These become high-priority objectives and influence the conduct of response forces, who are redirected toward security, enforcement, and traffic control tasks (12, 17). The looting myth also reinforces the perception of the need for centralized control and security-oriented management (27).

The myth of social breakdown also encompasses the assumption of widespread crime, and therefore, its influence exceeds that of the looting myth. The belief behind this myth is that society may deteriorate into chaotic and irrational behavior during a disaster. This perception highlights the fragility of human order and perceives the public as requiring stringent oversight and control, thereby legitimizing the use of coercive and enforcement measures to restore order (12, 23). This myth also serves as a basis for centralized governance (9, 21, 27) and for security-oriented management (26), with security and public order prioritized over humanitarian efforts (12, 18). In reality, authorities are often surprised by the public's calm, orderly behavior, characterized by unexpected initiatives and active engagement (6, 16). It is evident that the combination of looting, social disorder, disaster syndrome, and panic myths significantly shapes the operational patterns of authorities and event management (12).

The role-abandonment myth holds that officials will leave their positions during a disaster to care for their families. This assumption of widespread human failure leads to an operational response based on rigid systems, strict discipline, military-like images, and centralized hierarchy. The resultant response prioritizes control, enforcement, and centralized command over flexibility, initiative, and coordination, and allows limited preparedness for independent action by the public and civilian authorities as active participants in emergency plans (22, 27).

Similarly, the organizational paralysis myth assumes that organizations and civilian authorities will be unable to function effectively during a disaster. The response to this myth also prioritizes centralized, rigid, and hierarchical responses while reducing reliance on civilian bodies and local initiatives.

The myth of a lack of preparedness for donations and the reception of aid missions overlooks the need for coordination, screening, and logistical control, leading to significant strain on emergency systems, delays in the distribution of essential aid, and the waste of manpower and resources. As a result, authorities are forced to divert resources away from rescue, health services, and critical infrastructure to manage supplies or support aid missions that do not align with actual needs (18).

Table 3 presents the decision-making characteristics associated with each individual myth. However, there are also broader common characteristics that shape general responses. In this context, decision-making related to procedures, preparedness plans, and first responder training is influenced by the full set of myths, including how they are echoed in the media and how media coverage itself affects the decision-making process.

### 4.5 Findings from an integrative perspective on disaster events influenced by myths

The articles in the review encompass a range of disasters, including hurricanes and floods, earthquakes, terrorist attacks, bioterrorism events, aviation accidents, and humanitarian crises (2, 6, 9, 12, 22, 25, 29). However, despite differences in scale, cultural context, physical characteristics, and timing, we could detect a consistent pattern in how myths about public behavior affect the way the authorities mount a disaster response. Myths, such as fear of looting, the assumption that the public will react with panic, or the perception that the population is passive and burdens the rescue forces, may all lead to centralized management structures, widespread roadblocks, severe movement restrictions, and delayed public participation in response and recovery. Decisions focused on enforcement, control, and maintaining “order,” are often made at the expense of essential rescue and recovery activities, as seen after Hurricane Katrina (12, 29), the Christchurch and L'Aquila earthquakes (19, 25), and the Loma Prieta earthquake (28). Similarly, control of information, and suboptimal communication with the public due to fear of panic and social disorder, have been noted in events such as the anthrax attacks (2, 22).

The integrative approach clarifies that decision-making challenges stem not from the nature of the disaster, but rather from the influence of myth-based assumptions on judgments (16–18). When the decision-making relies heavily on myths, a pattern emerges in which authorities attempt to address myth-generated projections, rather than the actual situation and needs. Thus, the influence of myths extends beyond the boundaries of disaster type, culture, and context, into impacts on the nature of decisions and identifiable operational patterns.

These patterns not only fail to prevent the expected risks but also undermine the effectiveness of the response and, in turn, create additional harm that may worsen the consequences of the disaster and hinder survivors' ability to recover (2, 9, 22).

### 4.6 Recommendations for addressing the influence of myths on first responders' decision-making during disasters

Our findings suggest that addressing the influence of myths on decision-making necessitates a new conceptual framework. This will not merely correct protocols or day-to-day guidelines, but will also reshape the way emergency systems interpret social reality during a disaster. Research reports emphasize the need to move from a centralized, hierarchical approach grounded in disaster-related myths to an alternative approach grounded in empirical evidence and recognition of social complexity, which views information, flexibility, and local organization as essential resources rather than sources of risk. (20–23, 28). Within this framework, it is vital to re-examine decision-making processes, the way knowledge is generated and disseminated within emergency organizations, and the interactions between authorities, citizens, political bodies, and the media. The cumulative results suggest that reducing the influence of myths is not merely a matter of targeted modification but a paradigm shift toward a system that integrates empirical evidence, behavioral insights, and organizational structures that enable coordination, adaptation, and problem-solving in highly uncertain environments. The change should begin by recognizing the actual behavior expected during disasters as it manifests in reality (2, 6, 10, 17, 21).

#### 4.6.1 Training, professional education, and institutional embedding

Various reports emphasize the need to develop dedicated training programs, including assimilation processes and exercises, for first responders, emergency planners, aid organizations, volunteers, and senior officials. Such training should incorporate up-to-date, evidence-based knowledge about public behavior during disaster events, systematically refute misconceptions and myths, and present empirical findings demonstrating solidarity, cooperation, the public's capacity to function, and emergent citizen action (2, 6, 16, 17, 20, 21, 23).

#### 4.6.2 Adoption of alternative disaster management models

Literature evidence recommends shifting from hierarchical and centralized command-and-control models to alternative frameworks, such as the Emergent Human Resources Model (EHRM) (28), situational awareness and sensemaking models (16), problem-solving models (27), and community-based and participatory approaches (19). These models are flexible, support improvisation, and feature decentralized structures that encourage civic involvement through existing and emergent organizations, including local entities, while emphasizing coordination among the various bodies and actors involved (10, 16, 21).

#### 4.6.3 Paradigm shift: from viewing the public as a problem to viewing the public as a solution

One of the central recommendations in the literature is a shift in public perceptions of the role of the public during disasters. Instead of viewing citizens as a source of panic or a burden, they should be recognized as a key resource in response and recovery (9, 19–22). Research on community behavior during disasters reveals that citizens often act rationally and cooperatively, frequently providing first aid and life-saving assistance before official authorities (9, 16). Implementing this perspective requires updating procedures, establishing mechanisms for collaboration with unaffiliated volunteers, and institutional recognition of their contribution as an integral part of the national emergency system (23, 27).

#### 4.6.4 Open, consistent, and empowering communication with the public

It is recommended to adopt transparent, honest, and clear communication strategies that explain the known situation to the public, emphasize the importance of recommended actions, and highlight their contribution to personal and community safety. This strategy will enable trust-based information flow as opposed to a protective, centralized approach focused on preventing “panic” (3, 10, 18, 25, 26).

#### 4.6.5 Structural and institutional reforms

More broadly, our findings point to the need for structural reforms in emergency management systems. Among the recommendations are the establishment of joint coordination centers for all those involved in disaster response, including the local community (17). Other recommendations include establishing coordination mechanisms to prevent unilateral actions and improve cooperation between authorities and insurance companies, thereby preventing uncoordinated demolitions that harm insurance processes and the economic resilience of residents and businesses (25). Additional proposals include developing a dedicated role for managing “zero responders”, namely members of the public who act immediately at the onset of an event and carry out initial rescue, assistance, and life-saving actions (16). This will include improving warning systems and missing-persons documentation (9), as well as local-level recommendations such as examining the separation of FEMA from DHS (24).

In parallel, legislation and regulatory frameworks must be adapted to avoid reliance on mythical assumptions and to enable effective coordination and responsibility-sharing with emergent groups (6, 16, 22, 24). This will require a system-wide capacity to integrate, manage, and support emergent groups, and to update emergency plans according to current empirical findings (2, 16, 23, 27).

#### 4.6.6 Future research and development

Alongside the practical recommendations, literature reports highlight a clear need to deepen research and strengthen the connection between research, practice, and development in the field. Future empirical studies are required to examine the effectiveness of processes designed to reduce the influence of a belief in myths (16, 27).

## 5 Limitations

The present scoping review was designed to comprehensively and integratively map the existing body of knowledge, using broad search terms related to disaster myths and their influence on first responders' decision-making. Accordingly, the review did not aim to examine each disaster myth in isolation, but rather to identify recurring patterns of influence across different myths, disaster types, and response contexts. While this approach enables the identification of systemic and multi-level mechanisms through which myths shape decision-making, it limits the ability to assess the relative influence of individual myths separately. In addition, most of the studies included in this review were conducted in high-income countries, primarily in Europe, North America, Japan, and Australia. Therefore, the findings may not fully reflect the realities of low- and middle-income countries or other Global South contexts.

## 6 Conclusions

The results of this systematic review identify a significant influence of belief in myths on the decision-making of emergency authorities and first responders. The findings reveal that these beliefs are not merely theoretical but have tangible consequences for life-saving efforts, the extent of human suffering, and public safety, as they affect planning, management, and responses during emergencies. The review also identifies a lack of empirical studies linking beliefs in disaster myths to actual operational behavior in the field. This is a critical research gap that highlights the need for further research, including on the influence of social media and contemporary information environments on these relationships. More so, the present review found no empirical studies that examined whether implementing these recommendations actually changes existing perceptions or leads to different decision-making patterns during disasters. This gap highlights a clear need for further empirical research to examine how to reduce the impact of erroneous beliefs on disaster-related decision-making. Furthermore, the impact of belief in disaster myths may vary across political, cultural, and institutional settings. Therefore, future research should examine the influence of belief in disaster myths on first responders' decision-making in more diverse geographical, political, institutional, socio-economic, and Global South contexts.

## Data Availability

The original contributions presented in the study are included in the article/Supplementary material, further inquiries can be directed to the corresponding author.
