# Supplementary material for: The influence of belief in disaster myths on first responders' decision-making in emergencies and disasters — A scoping review
Source: Front Public Health. 2026 Jul 2;14:1801679. doi: 10.3389/fpubh.2026.1801679 (PMC13373037; doi:10.3389/fpubh.2026.1801679)
Supplement: Supplementary file 1 [file Data_Sheet_1.pdf]

Supplementary Table S1. Characteristics of Included Studies Included in the Review

Authors & Year	Citation No.	Type of Item	Purpose	Myths Discussed	Summary
Drury et al., 2013	2	Peer-reviewed journal article- Quantitative survey (N = 448)	To examine belief in myths among police, stewards, safety officers, and the public, and how myth-belief relates to coercive vs. participatory strategies.	Mass panic; anarchy/looting; helplessness	Belief in disaster myths increases support for coercive and paternalistic policies, whereas beliefs in public resilience promote collaborative and democratic policies that leverage community strengths. The belief in myths of panic, looting, and disorder among professionals, justifies, the use of coercion, information withholding, and exclusive expert control. Fear of public panic also leads to restricting information and concealing risk events. In contrast, beliefs in public resilience strengthens support for cooperation, shared responsibility, and reliance on citizen-led initiatives.
Carter & Amlôt, 2016	3	Peer-reviewed journal article - Theoretical	Examines how Social Identity Theory explains crowd behavior in emergencies and how this understanding can guide practical recommendations for better communication and incident management.	Panic, disorder; discusses disaster myths / misconceptions in general.	The article, examined responses in CBRN events, and reported that the belief that crowds will panic or become disorderly leads to ineffective decisions: withholding information, lack of transparent communication, increased control measures, and over-reliance on authority. Assuming public irrationality promotes a Command-and-Control approach, reduces public compliance, prolongs the incident, and creates unnecessary strain on resources.
Lorenz et al., 2018	6	Peer-reviewed journal article - Mixed-methods (observations, survey N=1,006, interviews)	To examine how disaster myths hinder cooperation between professional rescuers and unaffiliated responders.	Panic, disaster shock, looting, helpless-victim myth	The findings show that myths about public behavior reinforce a hierarchical model that excludes civilians from the response, even when they have a genuine capacity to contribute. As a result, emergency plans struggle to incorporate informal civilian responses. Key barriers include mistrust, lack of communication and coordination mechanisms, and the failure of emergency services to recognize the potential and assistance that civilians are already providing. Consequently, unaffiliated responders are pushed into the role of "spectators" instead of being integrated as part of the overall response effort.
der Heid, 2004	9	Gray literature- Theoretical	To identify common misconceptions	Panic; “disaster syndrome”;	The article shows that emergency planning based on myths—particularly the panic myth and the looting myth—leads to

			about disasters and show how they lead to ineffective responses.	non-compliance; looting; dependency; total control; volunteers	ineffective responses. These false beliefs cause delays in warnings, reliance on a rigid command-and-control model, misallocation of resources, and disregard for public capabilities. As a result, clear alerts are avoided due to fear of inducing panic, decisions favor control over rescue, evacuations are delayed under the assumption that people will "flee on their own," coordination between teams deteriorates and hospitals become overloaded, and the public is treated as a problem rather than a resource, undermining rescue and assistance efforts. Ultimately, these myths shape emergency policies and practices in ways that worsen rather than reduce harm.
Quarantelli, 1986	10	Conference interpretation (grey literature)-Theoretical	To expose gaps between disaster planning and real management, and how mythical assumptions harm response.	Panic; chaos/anarchy; looting; mass flight; dependency; role abandonment	The article shows that myths about the public lead to centralized, artificial, and reality-detached planning, which produces poor communication, mismatched operational pathways, inter-organizational power struggles, and delays in decision-making — thereby weakening real managerial capacity during a disaster.
Tierney et al., 2006	13	Peer-reviewed journal article-Theoretical	To examine how the media disseminated disaster myths during and after Hurricane Katrina.	Looting, social breakdown, panic, mass flight, helplessness	Belief in myths led response agencies to adopt "law and order" policies, focusing on policing and neglecting rescue operations.
Imperiale & Vanclay, 2019	17	Peer-reviewed journal article-Qualitative (participant observation, communication analysis, retrospective interviews)	To examine how myths and command-thinking affected decisions in L'Aquila, showing systemic harm.	Public helplessness; panic; looting/anarchy	Disaster myths give rise to centralized institutional actions, including the establishment of cordons and exclusion zones, intensified identification checks, restricted movement of survivors and relatives, exclusion of the public from decision-making processes, heightened control, and suppression of spontaneous initiatives. Decisions tend to favor power consolidation, the use of emergency authorities, and the imposition of restrictions—based on the assumption that the public is incapable of coping. These decisions are shaped by viewing the public as a "risk" rather than a resource, thereby directing operational norms in the field.
Shrestha et al., 2022	18	Peer-reviewed journal article - A comparative qualitative study of extended post-earthquake cordon zones in Christchurch (2011) and	To analyze the establishment, management, and impacts of post-earthquake cordon zones (PECs) in two cities.	The public is generalized as "dangerous," "likely to interfere," or "likely to loot."	The myths directly influence governmental decisions, primarily through fear of looting and panic. This leads to the erection of extensive cordons, restricted access for residents, and increased reliance on armed forces. Large cordoned zones may be established to "keep interfering citizens away," and in some cases areas may be blocked without operational need. Law-and-

		L'Aquila (2009), based on 23 in-depth interviews with community stakeholders and emergency responders.			order models may marginalize emergency services and shift spatial management to security agencies, embedding long-term suspicion toward the public and undermining community recovery. Throughout the response and recovery phases, these myths actively shape how access boundaries are defined, how decisions are made, and how public space is managed.
Baker & Ludwig, 2018	19	Peer-reviewed journal article-Theoretical	To examine how institutions construct a "social threat" narrative to justify hierarchical command models and rigid disaster governance.	Public panic; social breakdown; public incapacity; unpredictable public behavior	Disaster myths become institutionalized "knowledge" and influence planning, training, resource organization, procedures, and protocols. They generate a hierarchical or militarized incident-management structure and affect the speed of decision-making and the quality of communication with the public. Such myths create operational patterns that are resistant to adaptation or improvisation and obscure the ability to view public behavior as a resource rather than a threat. First responders operate within an atmosphere of control and suspicion toward the public, which shapes the actual response on the ground (use of force, reduced flexibility, suppression of community initiatives).
Hodgson, 2020	20	Thesis (grey literature)-Qualitative document & case analysis (five major case studies)	To examine how agencies can adapt policies to recognize immediate responders and encourage bystander involvement.	Panic; social breakdown	Myths about public behavior (such as "panic" or "chaos") lead emergency organizations to form incorrect situational assessments, resulting in decisions that prioritize control, isolation, and distancing of civilians rather than collaboration, flexibility, and reliance on emergent responses. Consequently, resources are diverted toward order-maintenance instead of life-saving activities, and recognition of the potential role of bystanders as part of the initial response is delayed. This also reduces the essential flexibility required during the critical, early, chaotic phase of an incident.
Fischer, 1996	21	Peer-reviewed journal article-Theoretical literature review with case examples	To show gaps between myths and empirical findings and how "disaster mythology" harms coordination and decision-making.	Mass panic, irrational flight, looting, selfish/antisocial behavior, norm collapse ("anomie"), dependency ("disaster shock")	Belief in disaster myths leads emergency personnel to base their responses on imagined problems, delay evacuation and public information, operate within poor communication and jurisdictional conflicts, rely on media reports instead of professional situational assessment, and adopt rigid, defensive response patterns that undermine flexibility and public engagement.

Nogami, 2018	22	Edited volume chapter - Theoretical	To refute three major myths and show how they lead to mismanagement and further harm.	Panic; crime; donations	Disaster myths influence the decisions of emergency role-holders in problematic and dangerous ways. Belief in myths of panic, crime, and donations distorts emergency response and leads to misguided decisions. Fear of panic results in delaying or withholding essential information and in diverting forces toward crowd control instead of rescue. The looting myth shifts resources from search and rescue to property protection. The donations myth creates logistical overload that distracts authorities from core emergency tasks. Ultimately, viewing the public as irrational promotes a hierarchical and force-oriented response, reduces community engagement, and weakens the overall effectiveness of emergency operations.
Herrick, 2009	23	Peer-reviewed journal article - Theoretical	To develop a typology explaining civic involvement in disasters and how state security perceptions shape acceptance/rejection of civic behavior.	Panic myth; disorder myth; helplessness	Institutional myth-based perceptions shape first responders' environment, limit collaboration, and produce system-driven, mismatched responses.
Drabek & McEntire, 2003	24	Peer-reviewed journal article-Theoretical	To analyze emergent phenomena in disasters, critique flawed assumptions (myths) in command-and-control models, and propose improved frameworks.	Panic; looting; social collapse	The article indicates that myths about public behavior continue to shape organizational models (such as command-and-control) that guide how emergency agencies operate. The mismatch between these models and actual on-the-ground realities creates difficulties in coordination, communication, and real-time management. Emergent phenomena introduce additional challenges (overload, boundary-crossing of authority, lack of standardization) that further burden organizational decision-making during response efforts, forming a problematic backdrop for operational performance.
Tierney, 2003	25	Edited volume chapter-Theoretical	To examine the panic myth and the assumption that disaster management requires hierarchical control, with evidence from post-9/11.	Panic myth; collapse of social order	Disaster myths allow organizations to justify strengthened authority and centralized control. Beliefs in public panic and danger legitimize information withholding, adoption of militarized command-and-control models, and exclusion of volunteers and communities from emergency processes. Instead of expanding participation, institutions narrow the professional space and redefine "expertise" in ways that reinforce their power while weakening community-based, civilian approaches.

Raisio et al., 2019	26	Peer-reviewed journal article - Qualitative study (200 Finnish representatives, interviews & group discussions)	To examine authority and organizational perceptions of spontaneous volunteers ("fourth sector") and the tension between control and integration.	Helplessness or lack of self-control	Myths lead emergency agencies to reject volunteers, reduce cooperation, erect trust barriers, and narrow the effectiveness of response. The results reflect a lack of training for joint action.
Sun, 2010	27	Peer-reviewed journal article - Theoretical	To examine how myths—especially looting—shaped U.S. law and policy framing emergencies as law-enforcement issues.	Looting; widespread violence	The myth of "looting and anarchy" after disasters distorts authorities' responses. The belief that the public will descend into violence diverts resources from rescue to enforcement, delays humanitarian assistance, and leads to excessive use of force. As a result, rescue efforts are slowed, public trust is damaged, and decisions are based on fear rather than empirical evidence.
Clarke & Chess, 2008	28	Peer-reviewed journal article-Theoretical	To examine "elite panic" and the harmful influence on disaster management and emergency systems.	Panic myth; looting/chaos myth; helplessness myth; top-down control myth	Elite panic produces information withholding, coercive directives, and barriers to coordination, limiting responders' judgment and reducing public cooperation.
Dynes, 1994	29	Peer-reviewed journal article - Theoretical	To identify problematic assumptions in conventional emergency planning models.	Social chaos; panic; antisocial behavior; fear of losing essential personnel	Belief in myths of panic and helplessness leads emergency organizations to favor control, force, and centralized authority—often accompanied by withholding information from the public and excluding volunteers. As a result, misguided decisions are made, such as diverting resources to maintaining "order" instead of conducting rescue operations.
Neal & Phillips, 1995	30	Peer-reviewed journal article-Theoretical	To compare Command & Control vs. Emergent Human Resources models and show how myth-based assumptions weaken response.	Social chaos myth; public incapacity	Myths generate doctrines that shape how first responders operate. They exert an indirect, systemic, and continuous influence on planning, doctrine, and disaster management: producing rigid procedures, centralizing authority, delaying response, suppressing initiative, and creating coordination failures between agencies and with emergent groups. These myths do not affect individual responders alone; rather, they shape the entire operational system, including the responders who function within structures built upon these myths.
Boin et al., 2019	31	Gray literature (ResearchGate) - Theoretical	To present a strategic analysis of government response to Hurricane Katrina and identify	Widespread looting; collapse of social order; severe violence	Rumors treated as facts shift priorities from rescue to enforcement, delaying life-saving actions and producing overreactions based on false information.

			failure mechanisms.		
